# Benzyl isothiocyanate induces reactive oxygen species-initiated autophagy and apoptosis in human prostate cancer cells

**DOI:** 10.18632/oncotarget.15643

**Published:** 2017-02-23

**Authors:** Ji-Fan Lin, Te-Fu Tsai, Shan-Che Yang, Yi-Chia Lin, Hung-En Chen, Kuang-Yu Chou, Thomas I-Sheng Hwang

**Affiliations:** ^1^ Central Laboratory, Shin-Kong Wu Ho-Su Memorial Hospital, Taipei, 111, Taiwan; ^2^ Division of Urology, Department of Surgery, Shin-Kong Wu Ho-Su Memorial Hospital, Taipei, 111, Taiwan; ^3^ Division of Urology, School of Medicine, Fu-Jen Catholic University, New Taipei, 242, Taiwan; ^4^ Department of Urology, Taipei Medical University, Taipei, 111, Taiwan

**Keywords:** benzyl isothiocyanate, reactive oxygen species, apoptosis, autophagy, prostate cancer

## Abstract

Benzyl isothiocyanate (BITC) in cruciferous plants, which are part of the human diet, has been shown to induce apoptosis in various types of cancer. In this study, we show that BITC effectively suppresses the growth of cultured human prostate cancer cells (CRW-22Rv1 and PC3) by causing mitochondrial membrane potential loss, caspase 3/7 activation and DNA fragmentation. Furthermore, BITC induces ROS generation in these cells. The induction of apoptosis by BITC was significantly attenuated in the presence of N-acetylcysteine (NAC) and catalase (CAT), well-studied ROS scavengers. The induction of autophagy in BITC-treated cells were also diminished by the application of NAC or CAT. In addition, BITC-induced apoptosis and autophagy were both enhanced by the pretreatment of catalase inhibitor, 3-Amino-1,2,4-triazole (3-AT). Pretreatment with specific inhibitors of autophagy (3-methyladenine or bafilomycin A1) or apoptosis (Z-VAD-FMK) reduced BITC-induced autophagy and apoptosis, respectively, but did not abolish BITC-induced ROS generation. In conclusion, the present study provides evidences that BITC caused prostate cancer cell death was dependent on the ROS status, and clarified the mechanism underlying BITC-induced cell death, which involves the induction of ROS production, autophagy and apoptosis, and the relationship between these three important processes.

## INTRODUCTION

Epidemiologic studies continue to support that dietary intake of cruciferous vegetables may reduce the risk of various types of malignancies including prostate cancer [1, 2]. The anticancer effects of these vegetables have been attributed to isothiocyanates (ITCs) that are released upon chewing or during the maceration of certain cruciferous vegetables, in which ITCs are present as thioglucoside conjugates termed glucosinolates [3]. When the cruciferous vegetable is damaged, the enzyme myrosinase is released from a cellular compartment to hydrolyze the glucosinolates, producing ITCs and other products. Among the nearly 120 identified ITCs, benzyl ITC (BITC) is one of the best studied members. BITC has been shown to inhibit chemically induced cancer in animal models (reviewed in [4, 5], and to induce cell cycle arrest and/or apoptosis in various cultured cancer cell lines [6–15]. Although research over the past decade has shown that the molecular mechanism by which apoptosis is induced by BITC is complex and utilizes a wide range of signaling pathways that induce alterations including the expression of anti-apoptotic Bcl-2 family proteins, the activation of mitogen-activated protein kinases, the suppression of oncogenic signaling and the activation of caspases, the common link in apoptosis induction by BITC and other ITCs is the production of reactive oxygen species (ROS) [5]. The disruption of mitochondrial function and the activation of Bax were shown to be involved in BITC-induced ROS production, which ultimately led to the apoptotic death of cancer cells [7, 8]. It is also interesting that BITC, phenethyl ITC (PEITC) and sulforaphane (SFN) induce apoptosis in cancer cells but not in normal epithelial cells [8, 16, 17]. PEITC has been shown to differentially alter the expression of oxidative stress- and antioxidant defense-related genes in a prostate cancer cell line (PC3) and in a normal prostate epithelial cell line (PrEC) [18]; however, the mechanism underlying the differential sensitivity of cancer and normal cell types to apoptosis induced by ITCs remains unclear.

In addition to apoptosis, BITC, PEITC, and SFN induce autophagy, an evolutionarily conserved process for the bulk degradation of macromolecules, in various types of cancer cells. SFN was the first ITC to be documented to induce autophagy, resulting in the prevention of apoptosis induction by inhibiting the release of cytochrome c from mitochondria to the cytosol [19]. In our previous studies, we demonstrated that BITC induces protective autophagy via the inhibition of mTOR signaling [20]. In contrast, the induction of autophagy by BITC [21] or PEITC [22] leads to cell death in human breast and prostate cancer cells, respectively. Therefore, the role of BITC-induced autophagy may be cell type-specific, and the mechanism underlying the induction of autophagy by BITC warrants further investigation. Here, we showed that BITC effectively reduced cell viability in both hormone-sensitive (CWR22Rv1, Rv1) and hormone-refractory (PC3) human prostate cancer cell lines by disrupting the mitochondrial membrane potential (MMP), inducing caspase 3/7 activity and increasing DNA fragmentation, which are characteristics of apoptosis induction. Furthermore, we provide experimental evidence indicating that BITC-induced autophagy and apoptosis are both initiated by ROS.

## RESULTS

### BITC reduced cell viability via the induction of apoptosis in prostate cancer cells

**Figure 1:**
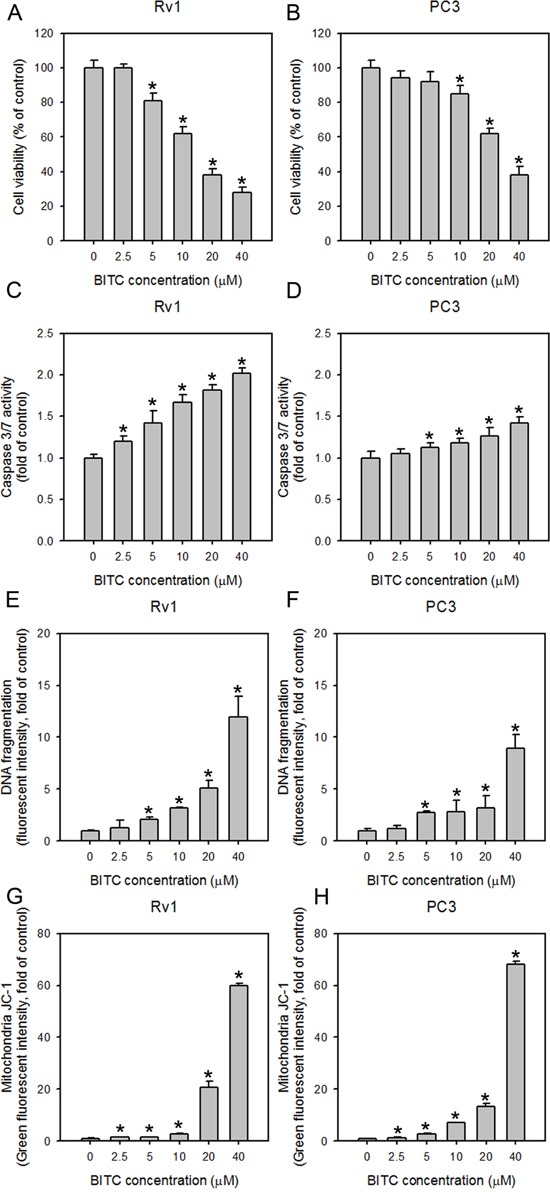
BITC treatment decreases cell viability, increases caspase 3/7 activity and DNA fragmentation, and disrupts the MMP in human prostate cancer cells The effect of BITC treatment on cell viability **A** and **B**. and caspase 3/7 activity **C** and **D**. in Rv1 and PC3 cells. The cells were treated with DMSO (control) or the indicated concentrations of BITC for 24 hrs. Cell viability and caspase 3/7 activity were assessed as described in the Materials and Methods section. The data represent the means of quadruplicate measurements for each condition, and the experiments were repeated three times. The results are presented as percentages of the control (means ± S.D.); *P<0.05. **E** and **F**., Effect of BITC treatment on histone-associated apoptotic DNA fragment release into the cytosol in Rv1 and PC3 cells. The cells were seeded in 6-well plates 24 hrs prior to the application of DMSO (control) or the indicated concentrations of BITC. The extent of DNA fragmentation was determined based on the incorporation of a FITC-conjugated antibody against BrdU, which labeled double-stranded DNA fragments in cells. The data shown represent triplicate measurements for each condition, and the experiments were independently repeated three times. The results are presented as the fold-change relative to the control (means ± S.D.); *P<0.05. **G** and **H**., BITC reduced the MMP in Rv1 and PC3 cells. Changes in the MMP in BITC-treated cells were revealed by flow cytometric analysis of JC-1 staining. Rv1 and PC3 cells were treated with DMSO (control) or the indicated concentrations of BITC for 24 hrs. The percentage of BITC-treated cells displaying decreased red fluorescence (indicating a reduction in the MMP and the disassociation of aggregated JC-1) or increased green fluorescence (indicating the collapse of the MMP and the formation of monomeric JC-1) compared to the DMSO-treated controls were measured by flow cytometry. The data shown represent triplicate measurements for each condition, and the experiments were repeated at least three times. The results are presented as the fold-change in green fluorescence intensity compared to the controls (means ± S.D.); *P<0.05.

The viability of Rv1 and PC3 cells, which represent hormone-sensitive and -refractory prostate cancer cells, respectively, was determined upon BITC treatment. BITC significantly inhibited the growth of both Rv1 and PC3 cells in a dose-dependent manner, as shown in Figure 1A and 1B. After 24 hrs of incubation, the viability of Rv1 and PC3 cells treated with 20 μM BITC was 38.01 ± 3.74% and 62.10 ± 3.21%, respectively, relative to the DMSO-treated controls. These results were compatible with our previous study, in which BITC exhibited higher toxicity to Rv1 cells than to PC3 cells [20]. The BITC-mediated reduction in Rv1 and PC3 cell viability was accompanied by the induction of apoptosis, as demonstrated by the dose-dependent activation of caspase 3/7 (Figure 1C and 1D) and increase in DNA fragmentation (Figure 1E and 1F; also see Supplementary Figure 1A for representative flow cytometry histograms). Several ITCs were reported to disrupt the MMP in cultured cancer cell lines [7, 23–26]. To understand the involvement of mitochondria in BITC-induced apoptosis, we detected the MMP in cells treated with BITC via flow cytometry following staining with the potential-sensitive dye JC-1. JC-1 dye that accumulates in the cytoplasm in the monomeric form emits green fluorescence in the absence of an intact MMP [27]. As observed in Figure 1G and 1H (the representative flow cytometry histograms are shown in Supplementary Figure 1B), BITC treatment for 24 hrs caused a statistically significant dose-dependent increase in the percentage of Rv1 and PC3 cells exhibiting green fluorescence, indicating collapse of the MMP. These data suggest that BITC induces the mitochondria-dependent apoptosis of human prostate cancer cells.

### BITC treatment induced ROS generation in prostate cancer cells

**Figure 2:**
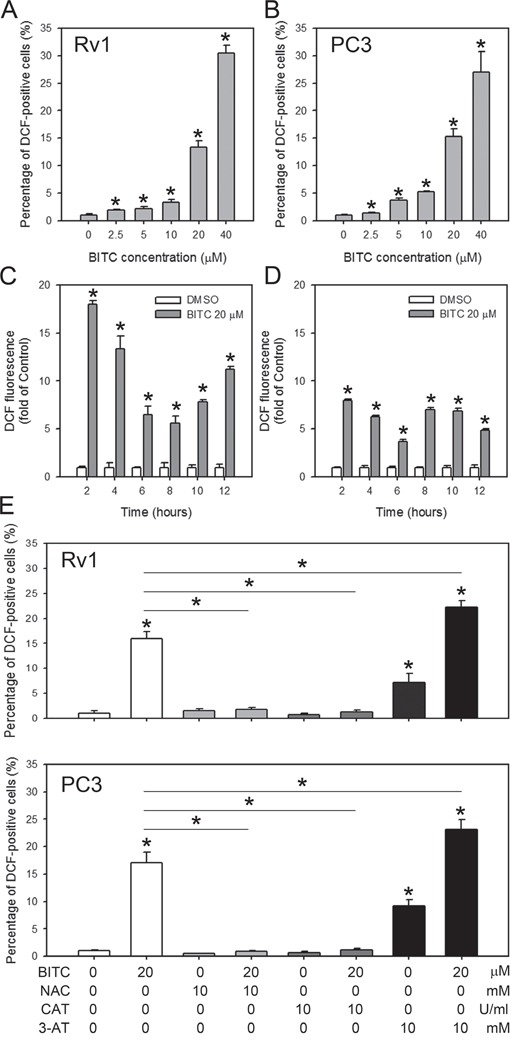
BITC induces dose-dependent, but not time-dependent, ROS generation in human prostate cancer cells Cells were treated with DMSO (control) or the indicated concentrations of BITC for 24 hrs **A** and **B**., or 20 μM BITC for the indicated period **C** and **D**., or pretreated 2 hrs with ROS scavengers, 10 mM NAC or 5,000 U/ml catalase (CAT), or catalase inhibitor, 3-AT **E**. in Rv1 and PC3 cells. Intracellular ROS generation was assessed via flow cytometry using H_2_DCFDA. The data were obtained from 10,000 events and were presented as the percentage of cells positive for DCF (green fluorescence in A, B and E) or the fold-change compared to the controls (in C and D). The values are expressed as the means ± S.D. of at least three independent experiments; *P<0.05.

Previous studies using a rat liver epithelial cell line have demonstrated that BITC-induced cell death is associated with the generation of ROS [26]. BITC was also shown to induce ROS in human breast cancer cells by targeting the mitochondrial respiratory chain [7, 8]. However, it is unclear if the BITC-induced cell death in prostate cancer cells is primarily initiated by ROS production. Therefore, we investigated the possible role of ROS generation in the BITC-induced apoptosis of prostate cancer cells. Intracellular ROS generation in the control (DMSO-treated) and BITC-treated Rv1 and PC3 cells was assessed via flow cytometry following staining with H_2_DCFDA, which specifically detects peroxides. H_2_DCFDA is cleaved by non-specific cellular esterases and is oxidized in the presence of peroxides to yield fluorescent 2′,7′-dichlorofluorescein (DCF). The results showed that BITC significantly induced ROS generation in a dose-dependent manner in human prostate cancer cells. In Rv1 and PC3 cells treated with 20 μM BITC for 24 hrs, the percentage of DCF-positive cells was 13.40 ± 1.09% and 15.28 ± 1.45%, respectively, relative to the DMSO-treated controls (Figure 2A and 2B; representative flow cytometry histograms in Supplementary Figure 2A). ROS production was detected as early as 2 hrs after the treatment of Rv1 and PC3 cells with 20 μM BITC. However, the generation of ROS in BITC-treated cells is not time-dependent (Figure 2C and 2D; representative flow cytometry histograms in Supplementary Figure 2B).

**Figure 3:**
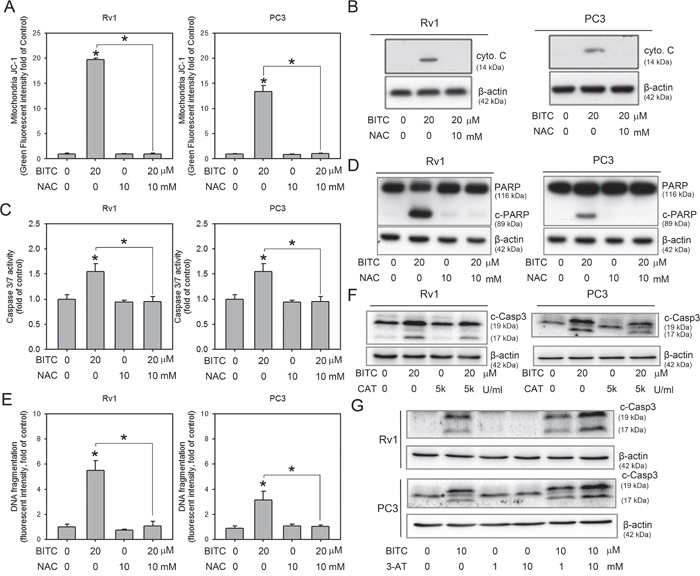
Inhibition of BITC-induced ROS attenuated apoptosis **A**. BITC-induced MMP collapse was rescued by NAC. Changes in the MMP in BITC-treated cells in the presence or absence of NAC were revealed by flow cytometric analysis of JC-1 staining. Rv1 and PC3 cells were treated with DMSO (control), 10 mM NAC, or 20 μM BITC with or without NAC for 24 hrs. The data shown represent triplicate measurements for each condition, and the experiments were repeated at least three times. The results are presented as the fold-change in the fluorescence intensity compared to the controls (means ± S.D.); *P<0.05. **B**. The BITC-induced increase in cytosolic cytochrome c was suppressed by NAC. The mitochondria-free cytosol protein fraction was prepared from Rv1 and PC3 cells subjected to the indicated treatment, and cytosolic cytochrome c and β-actin (loading control) expression was detected in these samples via Western blot as described in the Materials and Methods section. Representative samples from three independent experiments are shown. **C**. BITC-induced caspase 3/7 activation was suppressed by NAC. Rv1 and PC3 cells were treated with DMSO (control), 10 mM NAC, or 20 μM BITC with or without NAC for 24 hrs. Then, caspase 3/7 activity was assessed as described in the Materials and Methods section. The data shown represent the means of quadruplicate measurements for each condition, and the experiments were repeated three times. The results are presented as the percentages of the control levels (means ± S.D.); *P<0.05. **D**. BITC-induced PARP cleavage was diminished by treatment with 10 mM NAC. Total protein lysates were prepared from Rv1 and PC3 cells subjected to the indicated treatment, and of PARP and β-actin (loading control) expression was detected in these samples. Representative samples from three independent experiments are shown. c-PARP, cleaved PARP. **E**. The level of DNA fragmentation was assessed in Rv1 and PC3 cells treated with BITC with or without pretreatment with NAC. The data shown represent triplicate measurements for each condition, and the experiments were independently repeated three times. The results are presented as the fold-change compared to the controls (means ± S.D.); *P<0.05. **F**. Pre-treatment of catalase (CAT) suppressed BITC induced caspase-3 cleavage. Total protein samples from 20 μM BITC-treated cells with or without 2 hrs pre-treatment of 5,000 U/ml CAT were subjected to the detection of cleaved-caspase 3 (c-Casp3). Representative samples from three independent experiments are shown. **G**. Pre-treatment of catalase inhibitor, 3-AT, further enhanced BITC-induced apoptosis. Total protein samples from 10 μM BITC-treated cells with or without 2 hrs pre-treatment of 1 or 10 mM 3-AT were subjected to the detection of c-Casp3. Representative samples from three independent experiments are shown. * indicated a non-specific band detected in PC3 samples when using the same primary antibody as in Rv1 cells.

We next explored whether ROS production contributes to cell death (apoptosis) or survival (protective autophagy) caused by BITC in human prostate cancer cells. ROS generation in control (DMSO)- and BITC-treated Rv1 and PC3 cells with or without the ROS scavengers, NAC or CAT, was assessed by flow cytometry after staining with H_2_DCFDA. Consistent with the data shown in Figure 2A, treatment with 20 μM BITC increased the percentage of DCF-labeled Rv1 and PC3 cells (Figure 3E). However, pretreatment with NAC or CAT for 2 hrs decreased the green fluorescence in BITC-treated Rv1 and PC3 cells to the basal level (Figure 3E; representative flow cytometry histograms in Supplementary Figure 3A). These data suggested that the induction of ROS in the 20 μM BITC-treated Rv1 and PC3 cells was successfully suppressed by pretreatment with 10 mM NAC or 5,000 U/ml CAT for 2 hrs. To manipulate BITC-induced ROS for the subsequent experiments, we also monitored the ROS generation in BITC-treated cells with or without the pretreatment of 3-AT, a catalase inhibitor. As shown in Figure 3E, pretreatment of 3-AT enhanced BITC-induced ROS generation.

### Suppressing BITC-induced ROS diminished apoptosis induction

We next investigated whether ROS production contributed to the disruption of the MMP in BITC-treated cells. As shown in Figure 3A, the disruption of the MMP by BITC was completely attenuated by pretreatment with NAC (also see Supplementary Figure 3B for representative flow cytometry histograms). To test whether the BITC-mediated disruption of the MMP was accompanied by the cytosolic release of apoptogenic molecules, mitochondria-free cytosolic fractions were prepared from control and BITC-treated cells in the absence or presence of 10 mM NAC, and these fractions were subjected to immunoblotting for cytochrome c. As shown in Figure 3B, treatment of Rv1 and PC3 cells with 20 μM BITC for 24 hrs resulted in an increase in the cytosolic level of cytochrome c. Additionally, the BITC-induced release of cytochrome c was inhibited by pretreatment with NAC for 2 hrs prior to BITC application. Because the release of cytochrome c initiates the caspase cascade and leads to apoptosis [28], we next evaluated the activation of caspase 3/7 and the level of DNA fragmentation in BITC-treated cells with or without pretreatment with NAC. As shown in Figure 3C, pretreatment with NAC significantly inhibited BITC-induced caspase 3/7 activation. Full-length poly(ADP-ribose) polymerase (PARP) is a 116 kDa protein that is involved in DNA repair and chromatin structure formation. During apoptosis, PARP is cleaved by caspase-3, and possibly other caspases, into an 89 kDa fragment [29]. As shown in Figure 3D, cleaved PARP was clearly detected in BITC-treated Rv1 and PC3 cells, but the levels of cleaved PARP were diminished by pretreatment with NAC. BITC-induced DNA fragmentation was also suppressed by the inhibition of BITC-induced ROS production using NAC (Figure 3E; also see Supplementary Figure 4 for representative flow cytometry histograms). It is possible that NAC could react with ITC through thiol conjugation and therefore reduces cellular uptake of BITC by conjugating in the medium [30], we therefore pretreated the BITC-treated cells with CAT or CAT inhibitor, 3-AT to access the apoptosis induction. As shown in Figure 3F and 3G, pretreatment of 5,000 U/ml CAT or 10 mM 3-AT decreased or increased the cleaved caspase 3, respectively. Collectively, these results indicated that BITC-induced apoptosis is depend on the level of ROS generation.

### BITC-induced autophagy was dependent on ROS generation

**Figure 4:**
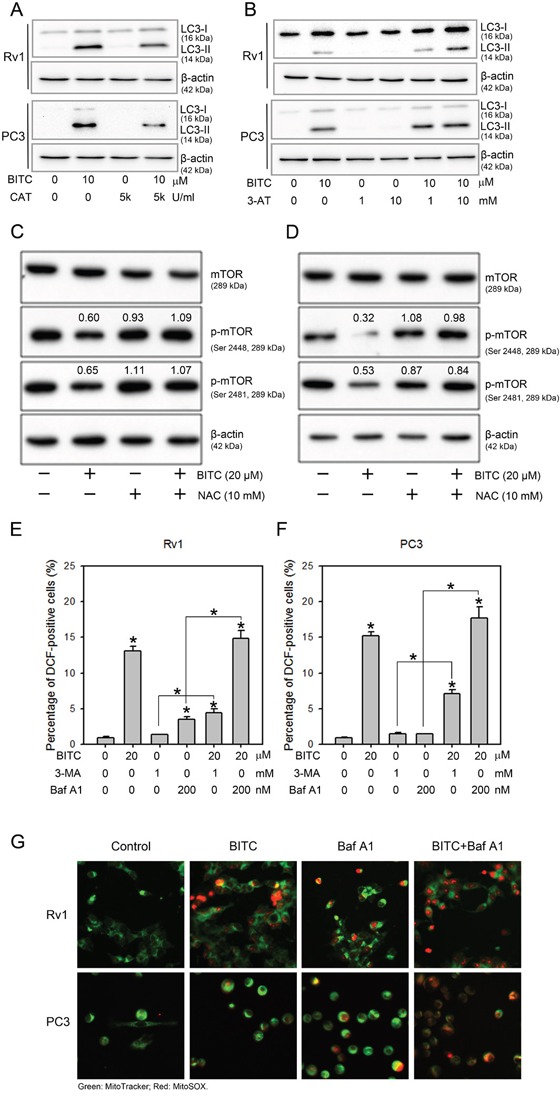
BITC-induced autophagy was attenuated by CAT or 3-AT, but autophagy inhibitors did not inhibit BITC-induced ROS production BITC-induced LC3-II processing were attenuated by pretreatment with CAT or 3-AT; and the inhibition of mTOR was recovered by pretreatment with NAC. Rv1 and PC3 cells were treated with DMSO (control), or 20 μM BITC with or without the pretreatment with 5,000 U/ml CAT, or 1 or 10 mM 3-AT, or 10 mM NAC for 24 hrs prior to harvesting. Total cell extracts were probed with antibodies against LC3 **A** and **B**., total mTOR (mTOR), phospho-mTOR (p-mTOR, Ser 2448) or phospho-mTOR (p-mTOR, Ser 2481) **C** and **D**. The expression level of β-actin served as a loading control. The relative expression levels of LC3-II, phospho-mTOR (Ser2448) and phospho-mTOR (Ser2481) were quantified via densitometric scanning, and the results are presented on above the bands as the fold-change relative to the DMSO-treated controls. Representative samples from three independent experiments are shown. Treatment with the autophagy inhibitors 3-MA and Baf A1 failed to suppress BITC-induced ROS production. ROS generation in Rv1 **E**. and PC3 cells **F**. treated for 24 hrs with DMSO (control), 20 μM BITC, 1 mM 3-MA, 200 nM Baf A1, or 20 μM BITC following pretreatment with 3-MA or Baf A1 for 2 hrs was assessed via flow cytometry based on H_2_DCFDA staining. The data were obtained from 10,000 events and were presented as the percentage of cells positive for DCF (green fluorescence) compared to the DMSO-treated controls. The values are expressed as the means ± S.D. of at least three independent experiments; *P<0.05. **G**. Merged fluorescence microscopic images (magnification x 200) of MitoSOX Red and MitoTracker Green staining in Rv1 and PC3 cells treated for 24 hrs with DMSO (control), 20 μM BITC, 200 nM Baf A1 or 20 μM BITC following pretreatment with 200 nM Baf A1 for 2 hrs.

Mounting evidence suggests an essential role of ROS in the activation of autophagy (reviewed in [31–33]). We previously demonstrated BITC-induced protective autophagy in human prostate cancer cells [20]. Here, we designed experiments to determine whether BITC-induced autophagy is mediated by ROS. The processing of the microtubule-associated protein light chain 3 (LC3) serves as a marker of the activation of the autophagy pathway [19]. Total protein samples from Rv1 and PC3 cells treated with DMSO or 20 μM BITC for 24 hrs were assessed for LC3-II formation using an LC3 antibody that detects both LC3-I and LC3-II (APG8b, N-term, Abgent). The LC3-II protein level was elevated in cells treated with 20 μM BITC but was diminished in BITC-treated cells pretreated with 5,000 U/ml CAT (Figure 4A). These results indicated that BITC-induced autophagy was suppressed by an antioxidative enzyme, which prevented BITC-induced ROS generation. In addition, when pretreated with 3-AT, which inhibits endogenous catalase activity, we detected increased autophagy in BITC-treated cells (Figure 4B). In our previous studies, we found that BITC-induced autophagy is accompanied by the inhibition of the mTOR signaling pathway [20]. We next investigated the effect of ROS suppression on the mTOR status in BITC-treated cells. As shown in Figure 4C, BITC-induced mTOR inhibition was attenuated by pretreatment with NAC.

### Inhibiting BITC-induced autophagy failed to suppress ROS generation

We next investigated whether BITC-induced autophagy contributes to ROS generation in prostate cancer cells. The inhibition of BITC-induced autophagy was achieved via pretreatment with 3-methyladenine (3-MA), an inhibitor of class III PI3-kinase [19], or bafilomycin A1(Baf A1), an inhibitor of the late phase of autophagy that blocks vacuolar H+ ATPase [34]. Consistent with our previous report [20], pretreatment with 3-MA or Baf A1decreased and increased the processing of the LC3-II protein, respectively, in BITC-treated Rv1 and PC3 cells (data not shown), indicating autophagic flux in prostate cancer cells upon BITC treatment. As shown in Figure 4E and 4F (also see Supplementary Figure 5 for representative flow cytometry histograms), the percentages of DCF-positive BITC-treated Rv1 and PC3 cells were 13.80 ± 0.60% and 15.20 ± 0.61%, respectively, compared to the DMSO-treated controls. In PC3 cells treated with 3-MA or Baf A1 alone, the percentage of DCF-positive cells was slightly increased. However, in Rv1 cells treated with 3-MA or Baf A1 alone, the percentage of DCF-positive cells was significantly increased to 3.50 ± 0.36% and 4.43 ± 0.55%, respectively, compared to the controls. In BITC-treated cells pretreated with either autophagy inhibitor, the generation of ROS was not affected. To further confirm the induction of ROS in BITC- and BafA1-treated cells, we stained Rv1 and PC3 cells with MitoTracker Green, a marker of functional mitochondria, and with MitoSOX Red, a fluorogenic red dye that specifically detects the production of superoxide by mitochondria. MitoSOX Red staining was detected in cells treated with BITC or Baf A1 and was increased in BITC-treated cells pretreated with Baf A1 (Figure 4G). Thus, the inhibition of autophagy did not prevent BITC-induced ROS generation in prostate cancer cells.

### Autophagy inhibitors increased the BITC-induced apoptosis in prostate cancer cells

**Figure 5:**
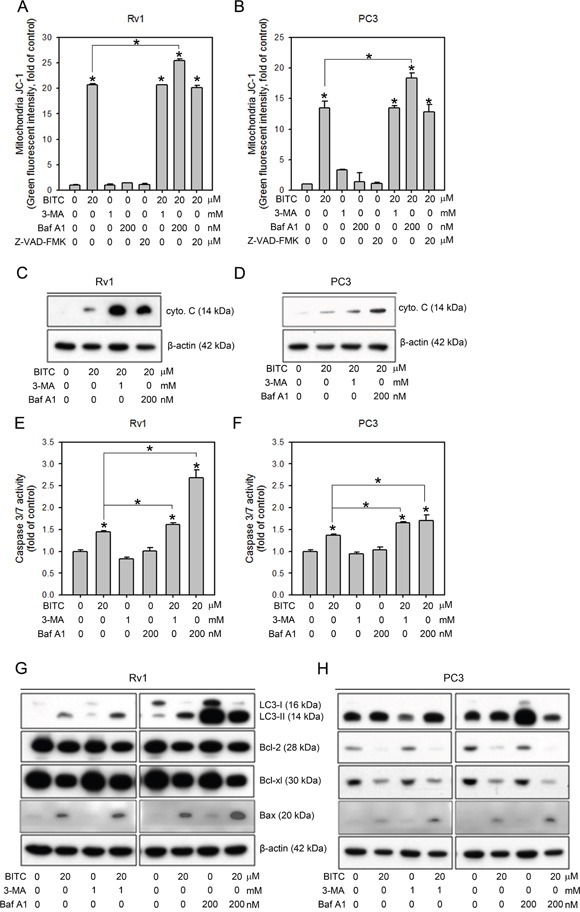
Inhibiting autophagy further increases BITC-induced apoptosis via the down-regulation of anti-apoptotic Bcl-2 family proteins **A** and **B**. Changes in the MMP were measured based on JC-1 staining in Rv1 and PC3 cells treated with 20 μM BITC with or without pretreatment with an autophagy inhibitor, 3-MA or Baf A1, or the caspase inhibitor, Z-VAD-FMK compared to the DMSO-treated controls. The data shown represent triplicate measurements for each condition, and the experiments were repeated at least three times. The results are presented as the fold-change in fluorescence intensity compared to the controls (means ± S.D.); *P<0.05. **C**. and **D**. Immunoblotting of the mitochondria-free cytosolic protein fraction from Rv1 and PC3 cells subjected to the indicated treatment. Representative samples from three independent experiments are shown. **E** and **F**. Caspase 3/7 activity in Rv1 and PC3 cells subjected to the indicated treatment was measured as described in the Materials and Methods section. The data shown represent the means of quadruplicate measurements for each condition, and the experiments were repeated three times. The results are presented as the percentage of the control levels (means ± S.D.); *P<0.05. **G** and **H**. Total protein lysates from Rv1 and PC3 cells subjected to the indicated treatment were used for immunoblotting for Bcl-2 family proteins, including the pro-apoptotic proteins Bax and Bad and the antiapoptotic proteins Bcl-2 and Bcl-xL. The level of β-actin served as a loading control. Representative samples from three independent experiments are shown.

We have previously demonstrated BITC-induced protective autophagy. Inhibiting BITC-induced autophagy via co-treatment with 3-MA resulted in increased sub-G0/G1 cell population and caspase 3/7 activities, leading to a further reduction in cell viability compared to BITC treatment alone [20]. However, whether autophagy inhibition disrupts mitochondrial function, affects the cytosolic release of apoptogenic molecules or alters the expression of anti- and pro-apoptotic proteins remained unclear. Therefore, we designed experiments to investigate the possible role of autophagy in BITC-induced apoptosis in our model. We first assessed mitochondrial membrane integrity in BITC-treated cells pretreated with the autophagy inhibitors 3-MA and Baf A1. As shown in Figure 5A and 5B (also see Supplementary Figure 6 for representative flow cytometry histograms), BITC-treated Rv1 and PC3 cells pretreated with 1 mM 3-MA disrupted the MMP to the same extent as BITC treatment alone. However, pretreatment with Baf A1 increased MMP disruption (from 20.69 ± 0.24- to 25.40 ± 0.40-fold in Rv1 cells and from 13.41 ± 1.14- to 18.31 ± 0.89-fold in PC3 cells relative to the controls). We also included Z-VAD-FMK to inhibit caspase activation, but the results showed that Z-VAD-FMK had no effect on MMP disruption in BITC-treated cells, indicating that the BITC-induced disruption of the MMP preceded the activation of caspase cascades. We next evaluated cytochrome c release in BITC-treated cells pretreated with autophagy inhibitors. The cytosolic protein fractions were extracted from cells treated with BITC alone or pretreated with 3-MA or Baf A1. These samples were subjected to immunoblotting for cytochrome c. As depicted in Figure 5C and 5D, inhibiting autophagy increased the release of cytochrome c in BITC-treated cells. Consistent with our previous studies [20], the activation of caspase 3/7 was further increased in BITC-treated cells pretreated with autophagy inhibitors, including 3-MA and Baf A1, in prostate cancer cells (Figure 5E and 5F). The Bcl-2 family proteins have emerged as critical regulators of both apoptosis and autophagy in response to diverse stimuli (reviewed in [35, 36]). To gain insight into the mechanism underlying BITC-mediated apoptosis and the effect of autophagy inhibition on BITC-mediated apoptosis, we compared the levels of Bc-2 family anti- and pro-apoptotic proteins in BITC-treated cells with or without pretreatment with autophagy inhibitors, i.e., 3-MA or Baf A1. As shown in Figure 5G and 5H, the expression levels of the anti-apoptotic proteins Bcl-xL and Bcl-2 were decreased in BITC-treated cells pretreated with autophagy inhibitors. These results indicated the involvement of altered expression of anti-apoptotic proteins in the execution of BITC-induced apoptosis after autophagy inhibition.

### BITC decreased cell viability in prostate cancer cells was depend on the ROS status

**Figure 6:**
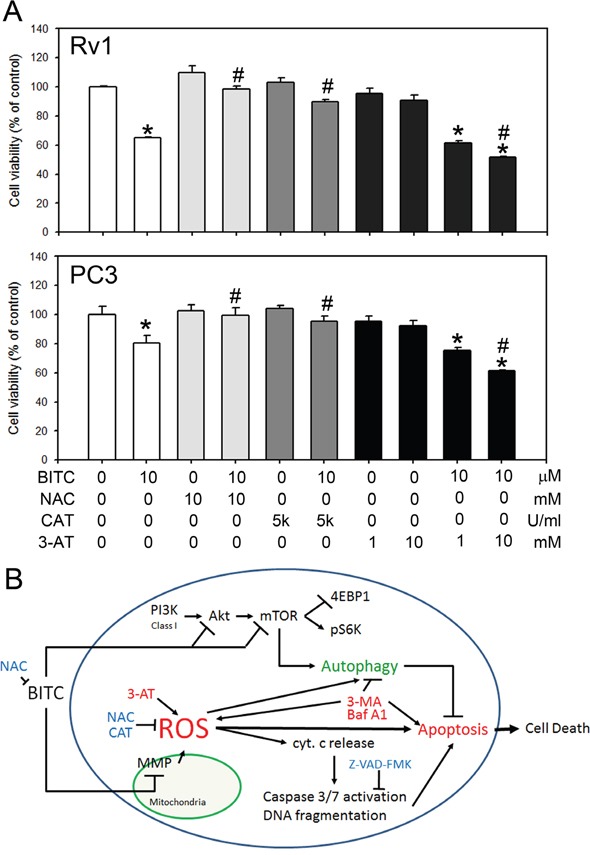
BITC decreased cell viability depended on the ROS status in prostate cancer cells **A**. Cell viability was detected in cells treated with BITC with or without the pre-treatment of 10 mM NAC, 5,000 U/ml CAT, or 1 or 10 mM 3-AT. The data represent the means of quadruplicate measurements for each condition, and the experiments were repeated three times. The results are presented as percentages of the control (means ± S.D.); *P<0.05 when compared to control; #P<0.05 when compared to BITC-treated cells. **B**. Schematic representation of a mechanistic model of the BITC-induced death of human prostate cancer cells, including the induction of ROS production, the collapse of the MMP, the induction of autophagy, the activation of caspase 3/7, and, ultimately, cell death. BITC induces ROS-initiated autophagy and apoptosis in human prostate cancer cells. BITC-induced autophagy was attenuated by the suppression of BITC-induced ROS production using NAC and CAT. BITC-induced autophagy and apoptosis was enhanced by a catalase inhibitor, 3-AT. Inhibition of BITC-induced autophagy enhanced apoptosis. However, the precise mechanisms by which ROS regulate autophagy induction and mTOR inhibition remain to be elucidated. cyt c, cytochrome c.

To determine if the ROS level in BITC-treated prostate cancer cells is vital for the induced cell death, we detected the cell viability in BITC-treated Rv1 and PC3 cells with or without the pretreatment of 10 mM NAC, which may neutralize BITC in the medium, 5,000 U/ml CAT, which suppressed BITC-induced ROS, and 1 or 10 mM 3-AT, which inhibits endogenous catalase activities. As shown in Figure 6A, BITC decreased cell viability was attenuated by NAC and CAT. In the other hand, pretreatment of 3-AT resulted in a dose-dependent decreased of cell viability in BITC-treated prostate cancer cells. These results were consistent with the observation that BITC-induced apoptosis is depend on the level of ROS generation. Collectively, our finding suggested that BITC induced apoptosis and cell death are depend on the status of cellular ROS level.

## DISCUSSION

Novel therapeutic approaches are needed to treat prostate cancer because it is the leading cause of newly diagnosed cancer, and is the second leading cause of cancer death in men [37]. Studies over the past decade have revealed that certain ITCs from cruciferous vegetables inhibit the growth of cultured cancer cells by inducing apoptosis. Among these ITCs, BITC was shown to induce the apoptosis of breast [6–8], pancreatic [10–12], and ovarian [13] cancer cells.

In the present study, we revealed that BITC effectively reduces the viability of prostate cancer cells, similar to other cellular systems [26, 38–41], via the induction of apoptosis, which is involved in the disruption of the MMP, the activation of caspase 3/7 and an increase in DNA fragmentation (Figure 1). For the first time, we report that BITC induces ROS generation in both hormone-sensitive (Rv1) and hormone-refractory (PC3) human prostate cancer cells (Figure 2). The concept of examining ROS generation in BITC-treated prostate cancer cells came from a previous study showing that ROS generation was induced by BITC in rat liver epithelial cells [26]. The induction of ROS generation has also been demonstrated in human breast [7], pancreatic [9], myeloma [42], osteogenic sarcoma [43] and promyelocytic leukemia cells [44]. Consistent with our results, ROS generation in BITC-treated DU-145 cells (another type of hormone-refractory human prostate cancer cells) accompanied by increased disruption of the MMP has been reported [24]. A dose-dependent increase in ROS generation was observed in both Rv1 and PC3 cells treated with BITC (Figure 2A and 2B), as demonstrated by the increased percentage of DCF-positive cells. Although a high level of ROS was detected as early as 2 hrs post-BITC treatment, BITC-induced ROS production was not time-dependent (Figure 2C and 2D). It is possible that pre-existing antioxidant enzymes act to relieve the ROS stress induced by BITC. To investigate the impact of ROS on BITC-treated cell death, NAC was utilized to suppress BITC-induced ROS accumulation initially according to previous reports [45, 46]. We found that pretreatment with 10 mM NAC for 2 hrs prior to the administration of BITC completely abolished BITC-induced ROS production (Figure 3). Because BITC-mediated ROS generation, MMP disruption, cytochrome c release, caspase 3/7 activation and cytoplasmic histone-associated DNA fragmentation were significantly attenuated in the presence of NAC (Figure 3), it is reasonable to conclude that the death of human prostate cancer cells caused by BITC was triggered by ROS generation. However, it is known that electrophilic ITCs is able to conjugate with NAC rapidly by forming thiocarbamates [47]. Thus, pre- or co-treatment of NAC may cancel the cellular responses to BITC as shown in a previous study working on PEITC [30]. We therefore utilized catalase (CAT) and catalase inhibitor, 3AT, to manipulate BITC-induced ROS and investigated the apoptosis induction. As shown in Figure 2E, ROS generation in BITC-treated cells was suppressed to basal level or further enhanced with the pretreatment of 5,000 U/ml CAT or 10 mM 3-AT, respectively. Accordingly, the apoptosis induction, judged by the level of cleaved caspase 3, in BITC-treated cells was decreased and increased with the pretreatment of CAT and 3-AT, respectively (Figure 3F and 3G). These results supported the idea that apoptosis induction is depend on the ROS level in BITC-treated prostate cancer cells. As mentioned above, several studies have shown that ROS generation is a critical event in the initiation of BITC-induced cell death. However, the precise mechanism by which ITCs promote ROS generation remains to be elucidated.

We previously reported that BITC induces protective autophagy in human prostate cancer cells via the inhibition of mTOR signaling [20]. Here, BITC-induced autophagy and mTOR inhibition were attenuated by pretreatment with CAT or 3-AT (Figure 4A and 4B), suggesting that BITC-induced ROS production initiates not only apoptosis but also autophagy. Accumulating studies have shown that ROS generation is related to the induction of autophagy (reviewed in [32]). ROS produced during nutrient starvation or hypoxia, stimulate autophagy via multiple signaling pathways. For example, the oxidation and inactivation of Atg4A by ROS enables the conjugation of LC3 to phosphatidylethanolamine, leading to the promotion of autophagy [48]. Furthermore, the knockdown of the basal expression of TP53-induced glycolysis and apoptosis regulator (TIGAR), which reduces the intracellular ROS levels, is sufficient to stimulate ROS production and protective autophagy induction in a mTOR- and p53-independent fashion [49]. ROS can also directly inhibit mTOR via the activation of TSC2 by ATM [50]. Although ROS are involved in autophagy induction, autophagy can exert a profound effect on ROS production. For example, the selective degradation of the H_2_O_2_ detoxifying enzyme catalase via autophagy results in increased lipid peroxidation and autophagic cell death in caspase 8-silenced cells [51]. In our model, the inhibition of BITC-induced autophagy using 3-MA or Baf A1 failed to suppress BITC-induced ROS generation (Figure 5E and 5F), suggesting that BITC-induced ROS generation is preceded by autophagy induction. Interestingly, the inhibition of autophagy using 3-MA or Baf A1 decreased and increased the ROS level in BITC-treated cells, respectively. It is possible that the inhibition of BITC-induced autophagy at different stages may result in the activation of distinct signaling pathways to cross-talk with ROS induction. However, the exact mechanism requires further investigation.

Recent observations indicated that autophagy and apoptosis are often induced by the same stimuli and are subject to complex regulatory and crosstalk mechanisms [51]. We previously showed that the inhibition of autophagy using 3-MA decreased the viability of BITC-treated prostate cancer cells, indicating a protective role of BITC-induced autophagy [20]. In this study, we further demonstrated that another autophagy inhibitor, Baf A1, increased the disruption of the MMP, the release of cytosolic cytochrome c, and the activity of caspase 3/7 in BITC-treated prostate cancer cells (Figure 5A-5F). Bcl-2 and Bcl-xL are anti-apoptotic Bcl-2 family members that bind to pro-apoptotic BH3 family proteins such as Bax and Bad to modulate cell apoptosis. Bcl-2 has been reported to regulate autophagy by sequestering Beclin 1, which is essential for the initiation of autophagosome formation [52]. The inhibition of autophagy in BITC-treated cells led to the decreased expression of Bcl-2 and Bcl-xL (Figure 5G and 5H), thus increasing the level of apoptotic cell death. These results indicated that inhibiting BITC-induced ROS-initiated autophagy enhances apoptosis, providing a rationale for further preclinical and clinical evaluations of the efficacy of combined treatment with BITC and autophagy inhibitors against prostate cancer. Finally, we provided evidences in this study that BITC-induced cell death was depend on the induced ROS level in prostate cancer cells (Figure 6A). Decreased cell viability in BITC-treated cells was attenuated by the pretreatment of NAC or CAT, while 3-AT further enhanced BITC-induced cell death.

In conclusion, the present study advanced a mechanistic model, schematically presented in Figure 6B, explaining the molecular network involved in BITC-induced ROS generation, apoptosis and autophagy in human prostate cancer cells. The translational implications of our findings are that the efficacy of BITC as a chemopreventive agent against prostate cancer may be improved or compromised in the presence of autophagy inhibitors or antioxidants, respectively.

## MATERIALS AND METHODS

### Cell culture and reagents

The human prostate cancer cell lines CWR22Rv1 (Rv1) and PC3 were obtained from the American Type Culture Collection (ATCC) and maintained as described previously [53]. Cell culture reagents including RPMI 1640 medium, a penicillin and streptomycin antibiotic mixture, fetal bovine serum, non-essential amino acids, sodium pyruvate and glutamine supplements were obtained from Invitrogen (Carlsbad, CA, USA). BITC (purity of ~98%) was purchased from Sigma (St. Louis, MO, USA). The stock solution of BITC was prepared at a concentration of 10 mM in DMSO, and aliquots were stored at -20°C. Each cell line was cultured at 37°C in an atmosphere containing 5% CO_2_. The cells were treated with the indicated concentrations of BITC, and the control cells received an equal volume of DMSO. The final concentration of DMSO was less than 0.1%. The catalase, catalase inhibitor (3-Amino-1,2,4-triazole, 3-AT), 5,5′,6,6′-tetrachloro-1,1′3,3′-tetraethylbenzimidazolylcarbocyanine iodide (JC-1), propidium iodide (PI), and 6-carboxy-2′,7′-dichlorodihydrofluorescein diacetate (H_2_DCFDA) were purchased from Sigma. The caspase 3 substrates (Z-DEVD)_2_-R110 were purchased from Bachem (Torrance, CA, USA). All other chemicals were purchased from Sigma unless otherwise specified.

### Cell viability assays

Cell viability following BITC treatment was assessed using the WST-1 reagent (Roche Diagnostics, Mannheim, Germany) according to the manufacturer's instructions. The proliferation assays were performed using the following reagents: the ROS scavenger N-acetylcysteine (NAC) and catalase (CAT), the catalase inhibitor 3-Amino-1,2,4-triazole (3-AT), the autophagy inhibitors 3-methyladenine (3-MA) and bafilomycin A1 (Baf A1), and the broad-spectrum caspase inhibitor Z-VAD-FMK (Promega, Madison, WI, USA). The cells were pretreated with 10 mM NAC, 5,000 U/ml CAT, 1 or 10 mM 3-AT, 1 mM 3-MA, 200 mM Baf A1 or 20 μM Z-VAD-FMK for 2 hrs and then treated with BITC in the presence of the specified inhibitor for the indicated duration. Cell viability was determined as the percentage of the controls. Each condition was assessed in eight replicate wells, and the data were obtained from at least three separate experiments.

### Detection of apoptosis

BITC-induced apoptosis was measured based on (a) the activation of caspase 3/7 and (b) the increase in DNA fragmentation. The activation of caspase-3/7 was assessed using the substrate (Z-DEVD)_2_-R110 (Bachem, Torrance, CA, USA) as described previously [54]. Briefly, cells were plated in 96-well plates and allowed to attach during overnight incubation. The cells were pretreated for 2 hrs with the indicated inhibitor and then treated with DMSO (control) or the indicated concentrations of BITC. Subsequently, the cells were directly lysed by adding caspase 3/7 assay buffer containing (Z-DEVD)_2_-R110 substrate, and the lysates were incubated at 37°C for 1 hr. The fluorescence intensity of the proteolytically released fluorophore R110 was then measured using a plate reader (Victor X2, PerkinElmer, Inc., Waltham, MA, USA) at an excitation wavelength of 485 nm and emission wavelength of 535 nm. The induction of DNA fragmentation in BITC-treated cells was detected using a BD Pharmingen™ APO-DIRECT™ Kit (Becton Dickinson, BD; NJ, USA) according to the manufacturer's instructions. DNA fragmentation was detected using a FACSCalibur flow cytometer, and the data were analyzed using CellQuest Pro software (BD, NJ, USA).

### Detection of the mitochondrial membrane potential

Changes in the MMP in BITC-treated cells were measured via flow cytometry using JC-1 as described previously [27]. Thirty minutes prior to flow cytometric analysis, JC-1 was added to the cells at a final concentration of 10 μM. The cells were then examined using a FACSCalibur flow cytometer, and the results were expressed as a FL-1 (530 nm) versus FL-2 (585 nm) dot plot (BD, NJ, USA). JC-1 displays dual fluorescence emission depending on the status of the MMP. JC-1 forms aggregates in cells exhibiting high FL-2 fluorescence, indicating a normal MMP. Loss of the MMP results in a reduction in FL-2 signal and a concurrent gain in FL-1 fluorescence as the dye shifts from an aggregated to a monomeric state. Therefore, the retention of this dye in cells can be monitored based on the increase in FL-1 fluorescence.

### ROS generation

Intracellular ROS generation in cells treated with BITC was measured via flow cytometry after staining with H_2_DCFDA as described previously [8]. Briefly, 2 × 10^5^ cells were plated in 6-well culture plates, allowed to attach overnight, and exposed to DMSO (control) or the indicated concentrations of BITC for the specified periods. The cells were stained with 1 μM H_2_DCFDA, and the fluorescence was measured using a FACSCalibur flow cytometer (BD). The data were analyzed using CellQuest Pro software (BD). In some experiments, the cells were pretreated for 2 h with NAC, CAT, 3-AT, 3-MA, Baf A1 or Z-VAD-FMK prior to BITC exposure and analysis of ROS generation. In some experiments, ROS generation in cells was assessed via fluorescence microscopy after staining with MitoTracker Green (Invitrogen) and MitoSOX Red (Invitrogen). In brief, the cells were seeded in chamber slides (Sigma) overnight and subjected to the indicated treatment for 24 hrs. The cells were then exposed to 1.5 μM MitoSOX Red and 100 nM MitoTracker Green for 20 min at 37°C, followed by fixation in 2% paraformaldehyde for 1 h at room temperature. After washing, the coverslips were mounted on slides using Prolong gold antifade reagent (Invitrogen). The cells were examined using a Zeiss AXIO Imager.A2 microscope equipped with an epifluorescence system and a CCD camera connected to a computer. Images were captured and processed using QCapture-Pro software (QImaging Corp., Surrey, Canada). All settings, including the exposure time for fluorescence imaging, were identical between the different experimental groups.

### Detection of autophagy

#### Immunoblotting for LC3

To perform immunoblotting analysis of LC3 expression, cells subjected to the indicated treatments were harvested and lysed, and the protein concentration was determined using a BCA protein assay (Pierce, Rockford, IL, USA). Proteins were separated via 14% sodium dodecyl sulfate polyacrylamide gel electrophoresis (SDS-PAGE) and were transferred to polyvinylidene difluoride (PVDF) membranes (Millipore, Billerica, MA, USA), followed by probing with antibodies against LC3 (#AP1802a, MAP1LC3B, Abgent, San Diego, CA, USA). Subsequent immunoblotting procedures were performed using a chemiluminescence process (Millipore) as per the manufacturer's instructions.

### Western blot analysis

The protein levels in cells were examined using Western blot analysis as described for immunoblotting for LC3. To detect cytochrome c release, the mitochondria-free cytosolic protein fraction was isolated using a mitochondria isolation kit obtained from Thermo Fisher Scientific (Rockford, IL, USA). Antibodies against cytochrome c, mTOR, phospho-mTOR (Ser2481), phospho-mTOR (Ser2448), PARP, Bcl-xL, Bad and Bax were obtained from Cell Signaling Technology (Beverly, MA, USA). Antibodies against Bcl-2 and β-actin were obtained from Sigma. The intensity of the immunoreactive bands was determined using GeneTools software (Syngene, Frederick, MD, USA) after scanning the developed films. The results are expressed as the means ± standard deviation (SD) of three independent experiments.

### Statistical analysis

All experiments were performed at least in triplicate, and the data are expressed as the means ± SD. The statistical significance of the differences in the measured variables between the treatment and control groups was determined using a Student's t-test; and the differences were considered to be significant at p<0.05.

## SUPPLEMENTARY FIGURES


