# Supplementary material for: Benzyl isothiocyanate induces reactive oxygen species-initiated autophagy and apoptosis in human prostate cancer cells
Source: Oncotarget. 2017 Feb 23;8(12):20220–34. doi: 10.18632/oncotarget.15643 (PMC5386757; doi:10.18632/oncotarget.15643)
Supplement: Supplementary file 1 [file oncotarget-08-20220-s001.pdf]

Benzyl isothiocyanate induces reactive oxygen species-initiated autophagy and apoptosis in human prostate cancer cells

SUPPLEMENTARY FIGURES

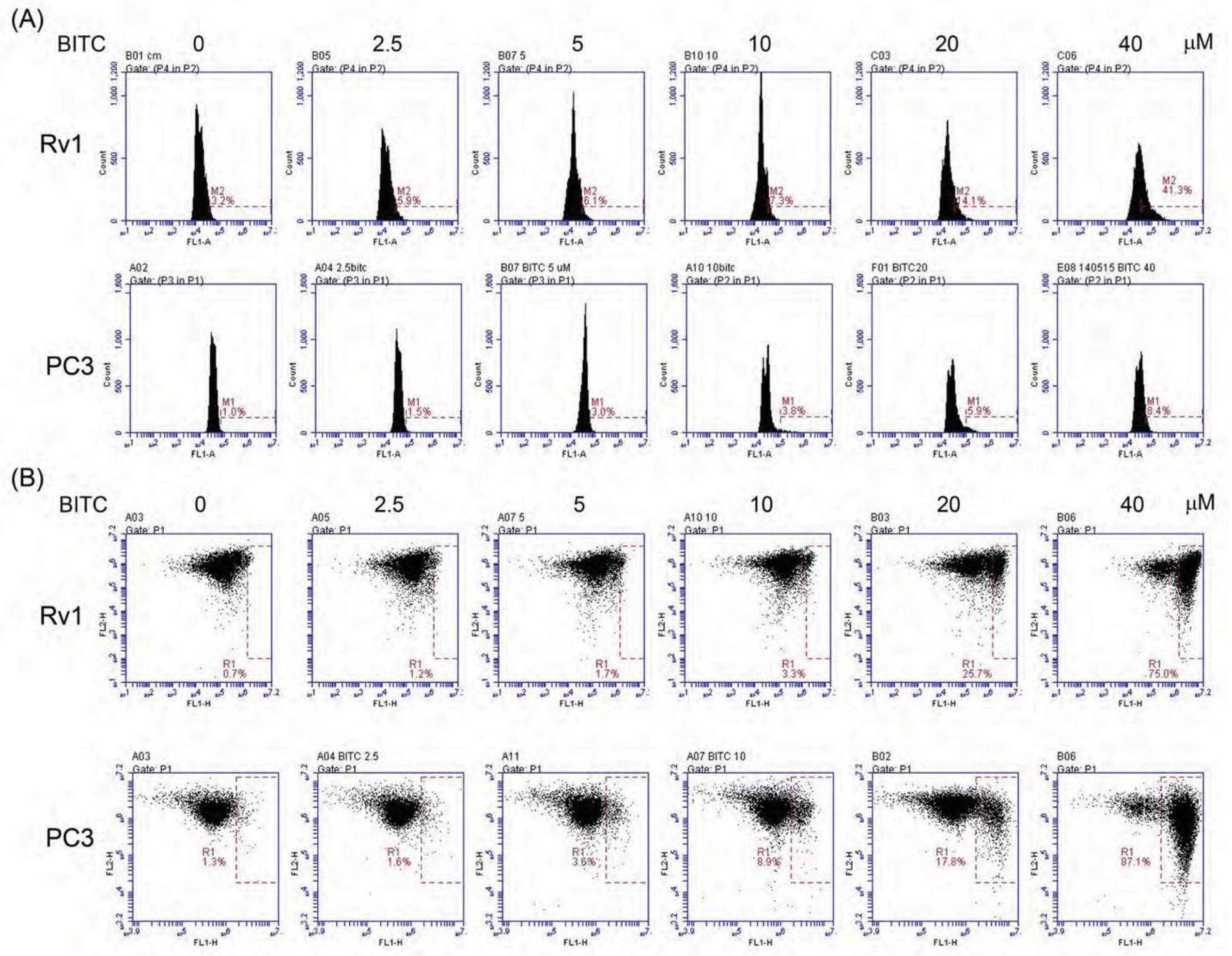

Supplementary Figure 1: Representative flow cytometry histograms of A. DNA fragmentation and B. mitochondria membrane potential (MMP) in Rv1 and PC3 cells treated with the indicated concentrations of BITC.

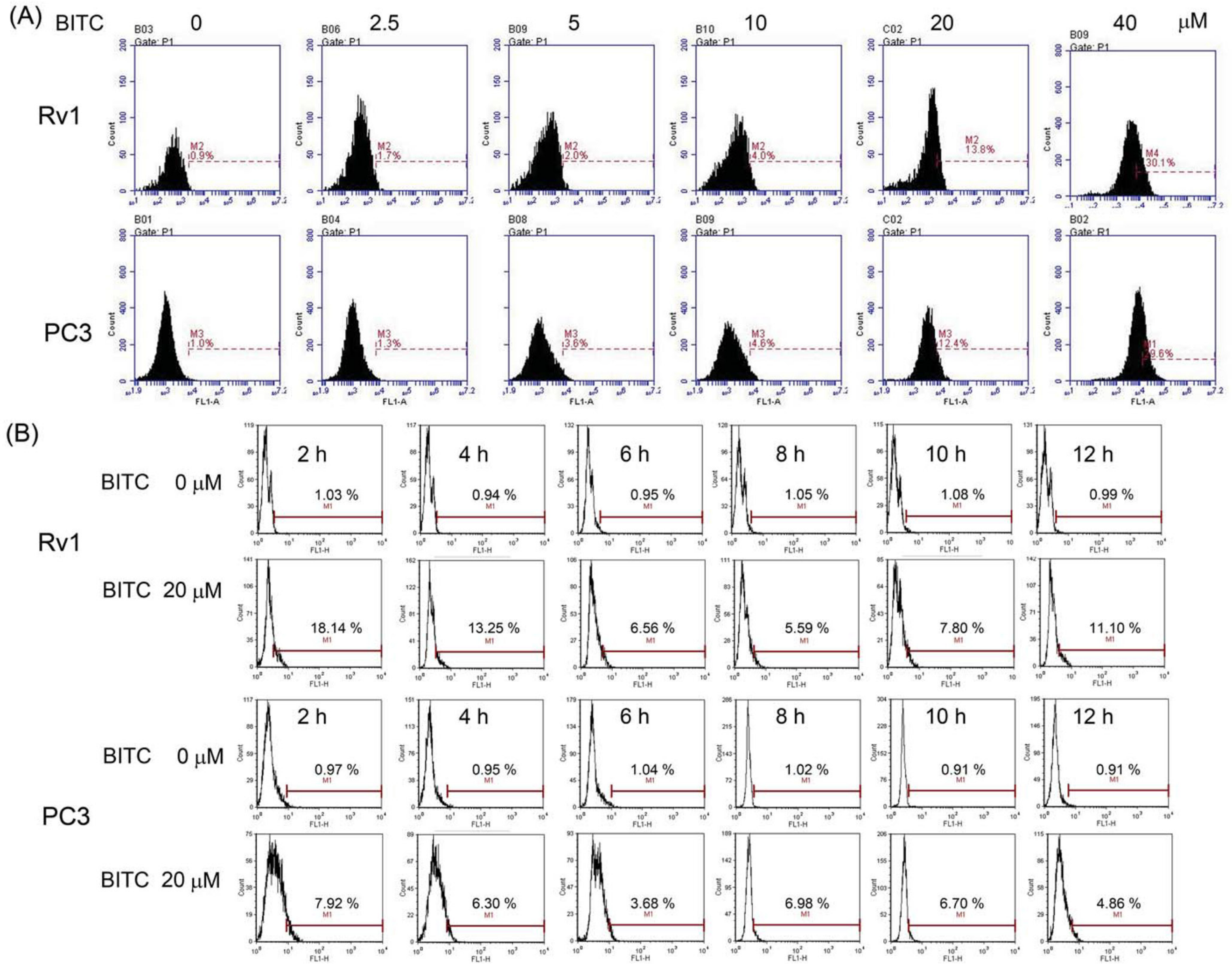

Supplementary Figure 2: Representative flow cytometry histograms of ROS generation indicated by DCF-positive Rv1 and PC3 cells treated with A. indicated concentrations of BITC for 24 hours and B. 20 µM of BITC for indicated duration of time.

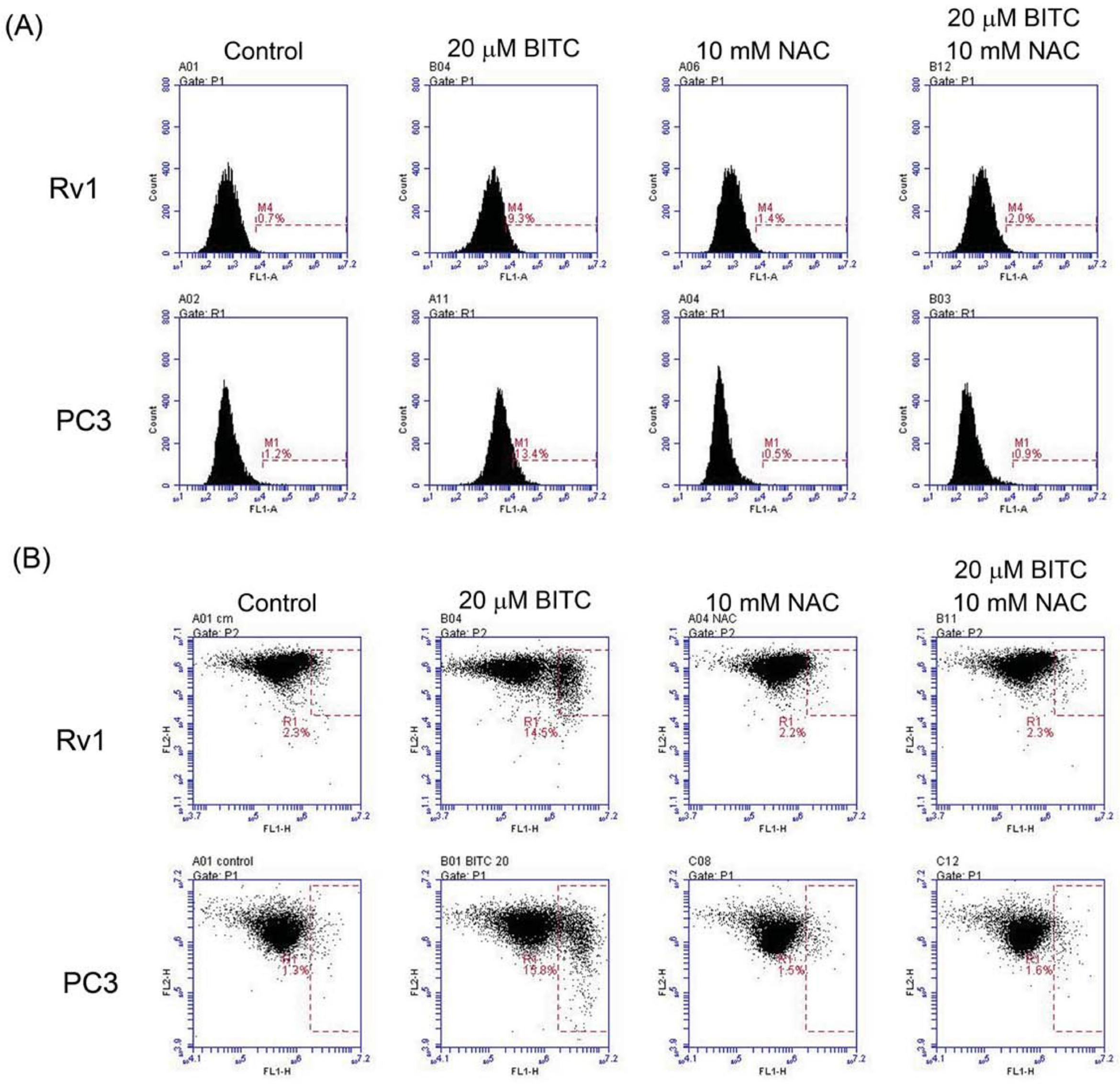

Supplementary Figure 3: Representative flow cytometry histograms of A. ROS generation indicated by DCF-positive cells and B. MMP in Rv1 and PC3 cells treated with 20 µM BITC for 24 hours with or without 2 hours pretreatment of 10 mM NAC.

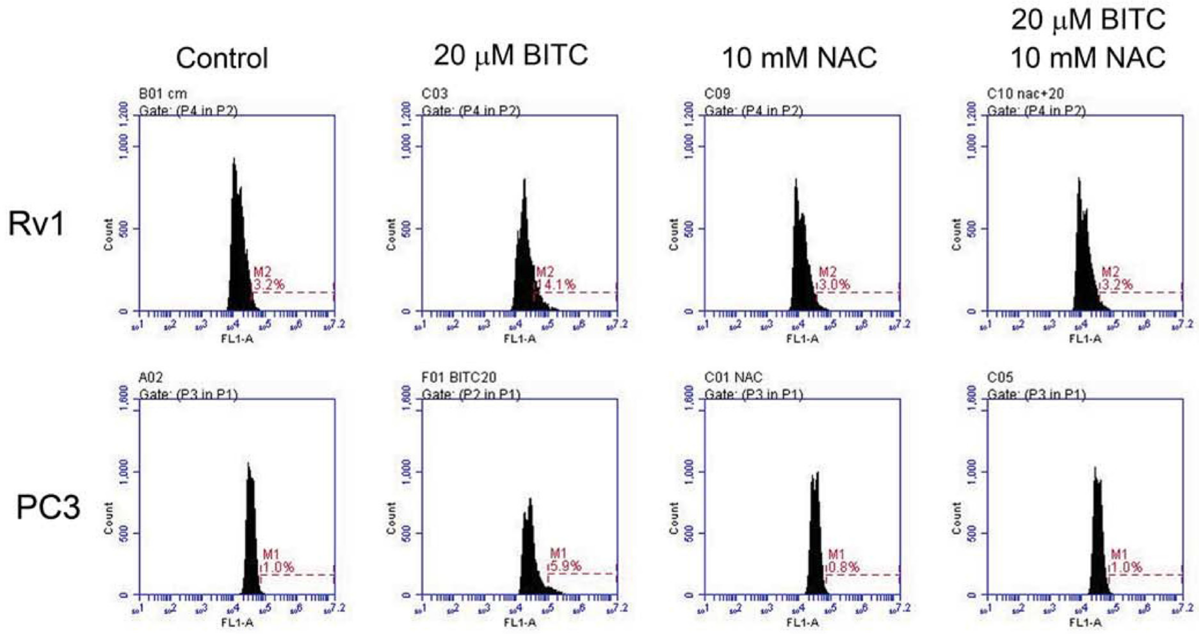

Supplementary Figure 4: Representative flow cytometry histograms of DNA fragmentation (TUNEL) in Rv1 and PC3 cells treated with 20 µM BITC for 24 hours with or without 2 hours pretreatment of 10 mM NAC.

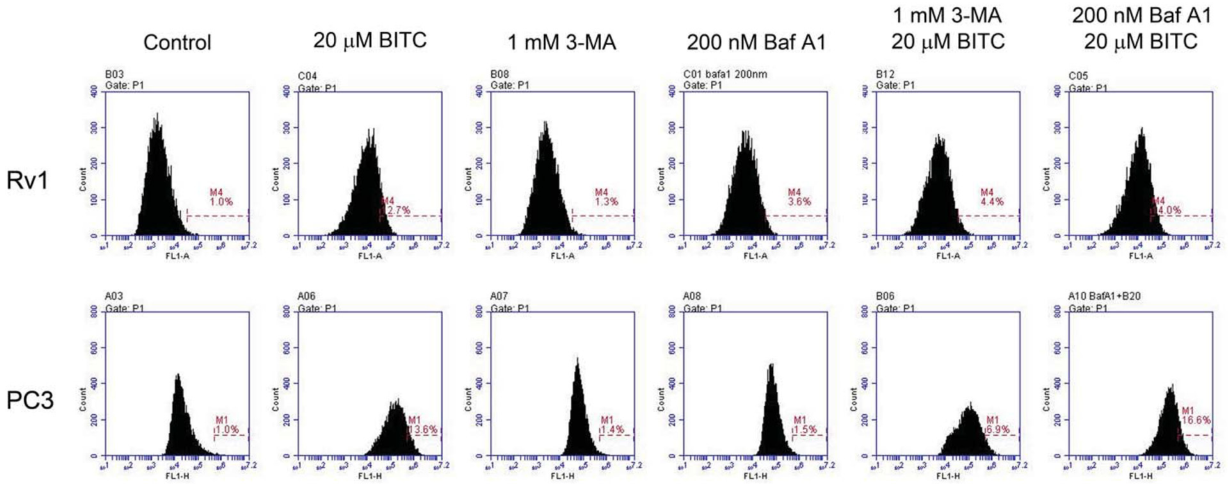

Supplementary Figure 5: Representative flow cytometry histograms of ROS generation in Rv1 and PC3 cells treated with 20 μM of BITC with or without 2 hours pretreatment of autophagy inhibitors, 3-MA or Baf A1.

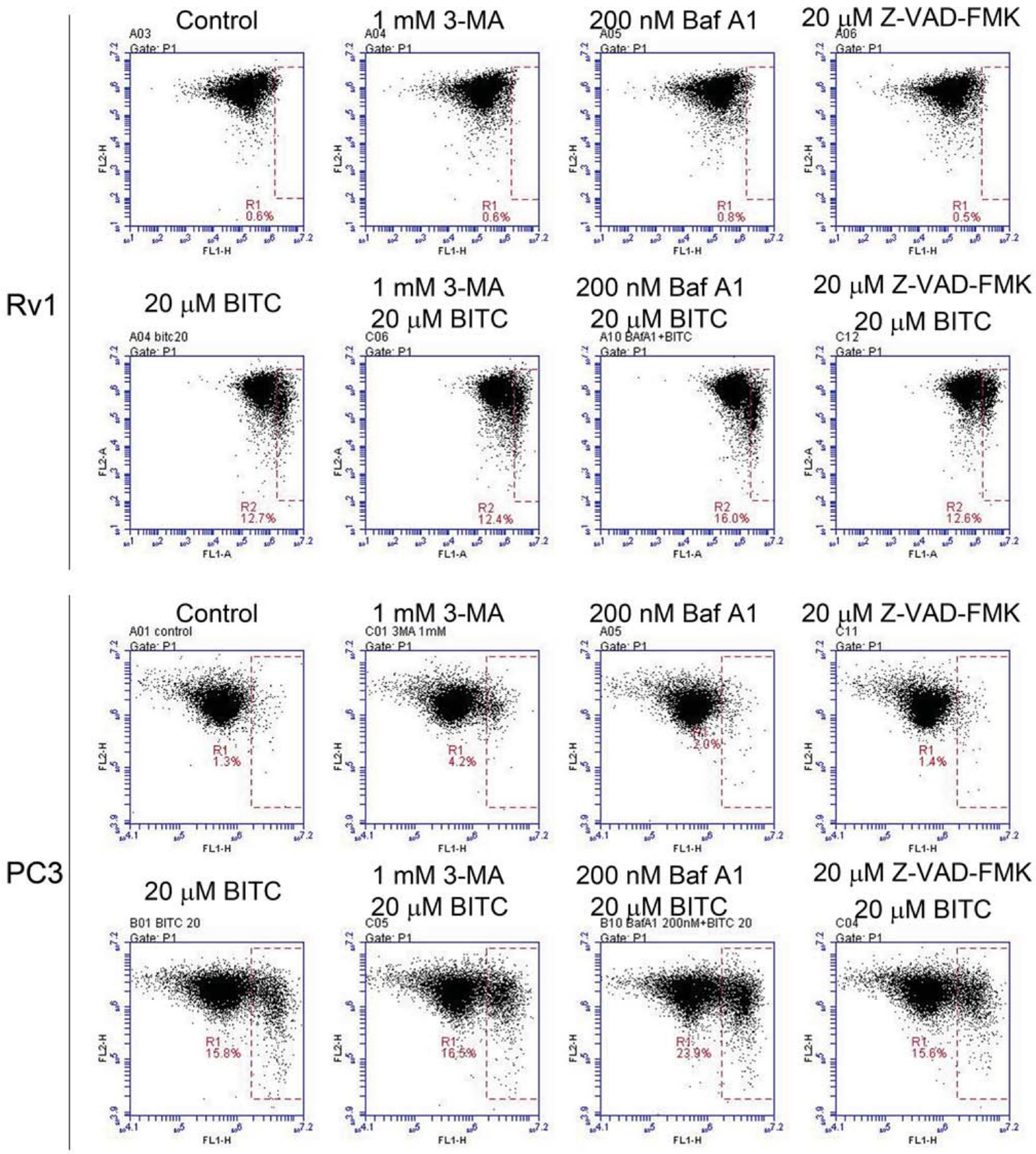

Supplementary Figure 6: Representative flow cytometry histograms of MMP disruption in Rv1 and PC3 cells treated with 20 μM BITC with or without 2 hours pretreatment of autophagy inhibitors, 3-MA or Baf A1, or caspase inhibitor, Z-VAD-FMK.
